# Effect of screw angulation on the bending performance of polyaxial locking interfaces: a micro-CT evaluation

**DOI:** 10.1038/s41598-023-48791-1

**Published:** 2023-12-08

**Authors:** Jakub Glowacki, Tomasz Bartkowiak, Piotr Paczos, Bartosz Gapinski, Patryk Mietlinski, Pawel Zawadzki, Weronika Weiss, Lukasz Lapaj

**Affiliations:** 1 Department of General Orthopaedics, Musculoskeletal Oncology and Trauma Surgery, Poznan University of Medical Sciences, Poznan, Poland; 2 Institute of Mechanical Technology, Poznan University of Technology, Poznan, Poland; 3 Institute of Applied Mechanics, Poznan University of Technology, Poznan, Poland

**Keywords:** Biomedical engineering, Mechanical engineering, Fracture repair

## Abstract

Polyaxial locking plates rely on a specific thread-to-thread interface of the screw head and the plate hole. The objective of this study was to evaluate the mechanical performance of single screw interfaces when inserted off-axis and to establish correlations between those parameters and the engagement of the screw head and the plate hole thread. Three polyaxial locking screw systems were inserted into the corresponding plates at various angles (0°, 5°, 10°, and 15° off-axis). The screws were tested until failure. A micro-CT was performed to examine the interface between the plate hole and the screw head. The standard insertion at 0° sustained the greatest maximum bending strength without relocation in the screw hole. Screws inserted at 15° showed a significant reduction in force of up to 44%, 55% and 57%, respectively. Micro-CT analysis of the interface showed a significant loss of thread engagement for off-axis insertion. Polyaxial plates offer additional advantages for off-axis placement of screws. However, this flexibility is related to a significant decrease in both thread engagement and bending strength compared to monoaxial insertion. Regardless the insertion angle, the loss of stability is comparable when screws are placed off-axis. Surgeons are advised to consider off-axis insertion as a salvage option, providing access to better bone stock.

## Introduction

The advancement of monoaxial locking plates has substantially enhanced surgical techniques, guaranteeing stability at the reduced fracture site through the rigidity of the locking construct^1,2^. Locking plate fixation is particularly beneficial in osteoporotic bones, yielding lower rates of implant dislocations and lower impairment rates to bone periosteum^3,4^. Unfortunately, the use of monoaxial plates requires a thorough surgical technique, preferably with the screw being locked in a perpendicular direction^3^. This requirement may result in positioning of the screw within poor bone stock or severely limit its length, especially in challenging periprosthetic fractures^5–7^. The application of monoaxial screws is also limited in periarticular fractures, where the requirement of inclining the screw is usually achieved with standard compression plates^2^. Clinical practice and biomechanical studies have demonstrated that only a precise insertion angle of the monoaxial screw can ensure proper fixation^8,9^. Kaab et al. stated that a deviation of more than 5° might lead to cross-threading of the screw head within the plate hole, significantly compromising stability^8^. As little as 5° to 10° off-axis insertion could reduce angular stability by 30% and 50%, respectively^8^.

Polyaxial designs theoretically guarantee a stable locking construct with off-axis screw positioning, potentially enabling surgeons to avoid osteoporotic areas, thus improving the purchase of the screw. Despite these substantial advantages over standard monoaxial constructs, several technical issues hindered its universal clinical use, as evidenced by observed clinical failures^10^. By now, a variety of polyaxial locking plates exists on the market^11^. Each manufacturer offers polyaxial plate systems with their own locking mechanism^11^. The hardware undergoes ASTM/ISO testing before reaching the market; however, manufacturers do not publicly disclose the exact stability of polyaxial locking interfaces^12^.

Furthermore, existing studies have predominantly evaluated the plates as complete constructs in fracture gap models^13–17^. Former studies performed on cadavers and sawbones, comparing polyaxial systems and standard monaxial plates, showed no significant differences in strength. In the cited studies, the authors claimed that they did not adhere to any specific standard. Although it may be assumed that there is some resemblance to a standard, such details were not provided by the authors of that work. The general standard for plates is the American Society for Testing and Materials (ASTM), ASTM F382-17, and for angled devices, it is ASTM F384-17^18,19^.

Only limited research focused on the mechanical properties of the screw head and plate hole interface^10,20^. However, to our best knowledge, none have comparatively tested the properties of locking interfaces of several variants within the same polyaxial plate system, using both destructive and non-destructive tests. Introducing a novel approach, microcomputed tomography (micro-CT) is a scientific method offering the establishment of overall connections and comprehensive examination of material microarchitecture^21^. It boasts advantages such as high resolution, effective visualization, and quantitative analysis of architecture related to the tested sample^21^. The three-dimensional reconstruction enables volumetric assessments and other more advanced measurements^22^.

This study aimed to analyze the extent of thread engagement between the screw head and the locking plate hole in the polyaxial plate construct using a micro-CT. The second objective was to test the mechanical properties of the ChM manufacturer’s 4.0, 5.0 and 7.0 ChLP polyaxial locking systems with screws positioned at various angles from 0° to 15°. We hypothesized that the amount of engagement would exhibit a significant correlation with the mechanical parameters of the interface.

## Material and methods

### Plates and screw placement

**Figure 1:**
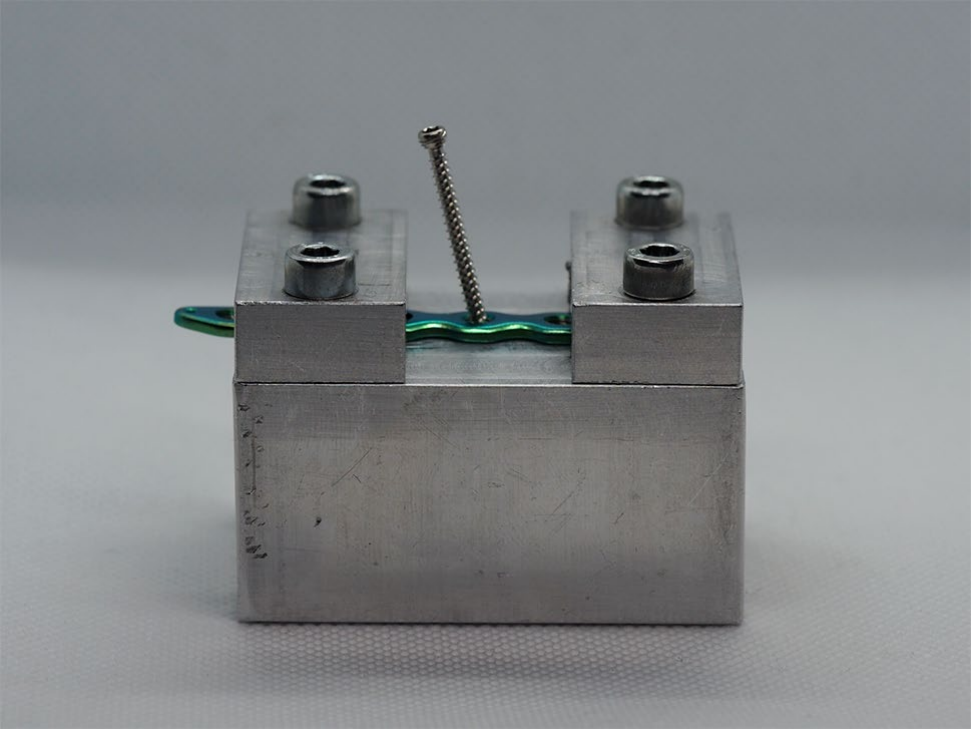
Pre-formed angulated block and 4.0 ChLP plate.

This study examined components from the 4.0, 5.0 and 7.0 ChLP polyaxial locking systems (ChM-manufacturer Sp. z o.o., Lewickie, Poland). The system consists of plates made of titanium alloy (ISO 5832-2/ASTM F67) and locking screws made of CoCrMo alloy (ISO 5832-12/ASTM F1537)^12,23,24^. The design allows for polyaxial screw insertion at an angle of up to 15° in all directions. The threads of the spherical screw head engage with the threaded part of the plate hole. In this study, the screws were inserted in three different configurations, using a pre-formed angulated block: at 0°, 5°, 10° and 15° (variance ± 1°) (Fig. 1). The insertion torque applied to each screw on each plate was standardized to the values recommended by the manufacturer, namely 1.0, 2.0 and 4.0 Nm for 4.0, 5.0 and 7.0 ChLP systems, respectively. The tightening was performed using the calibrated MicroClick MC 5 torque-limiting screwdriver by Proxxon Industrial (Föhren, Germany).

### Analysis of the thread-to-thread contact areas

Following the method developed by Kaczmarek et al. X-ray microcomputed tomography was utilized to measure the contact area between the threaded portions of the screw head and the plate hole^25,26^. Each construct was scanned using a metrological computed tomographic device (v|tome|x s240; GE Sensing & Inspection Technologies GmbH, Wunstorf, Germany). The X-ray beam power used for micro-CT scanning was 30.7 W (205 kV/150 μA), with a voxel size of 25.169 μm, and a per-image exposure time of 500 ms. Following the measurements, a 3D model of the threaded connection was generated, which was subsequently converted into a series of photos illustrating cross-sections taken every 20 μm orthogonal to the locking screw axis.

In these 2D images, thread engagement manifested as a region devoid of recognizable space between the screw and the plate. The contact or gap was identified through automated image postprocessing. For each image, it was assumed that full engagement occurred where no space existed between the screw and the plate. Each image received a score between 0 and 100% based on how much contact was determined by automatically examining the screw-plate interface region pixel by pixel. The average thread engagement (ATE) measure, which was calculated for all cross-sections, represented the arithmetic mean of the individual scores obtained for all analyzed images. The thread-to-thread contact regions of each screw head was visualized in a form of a three-dimensional polar plot. The image processing algorithm was implemented in Mathematica software (Wolfram Research, Champaign, IL, USA). More details about the method are described by Bartkowiak et al.^27^.

### Mechanical setup

**Figure 2:**
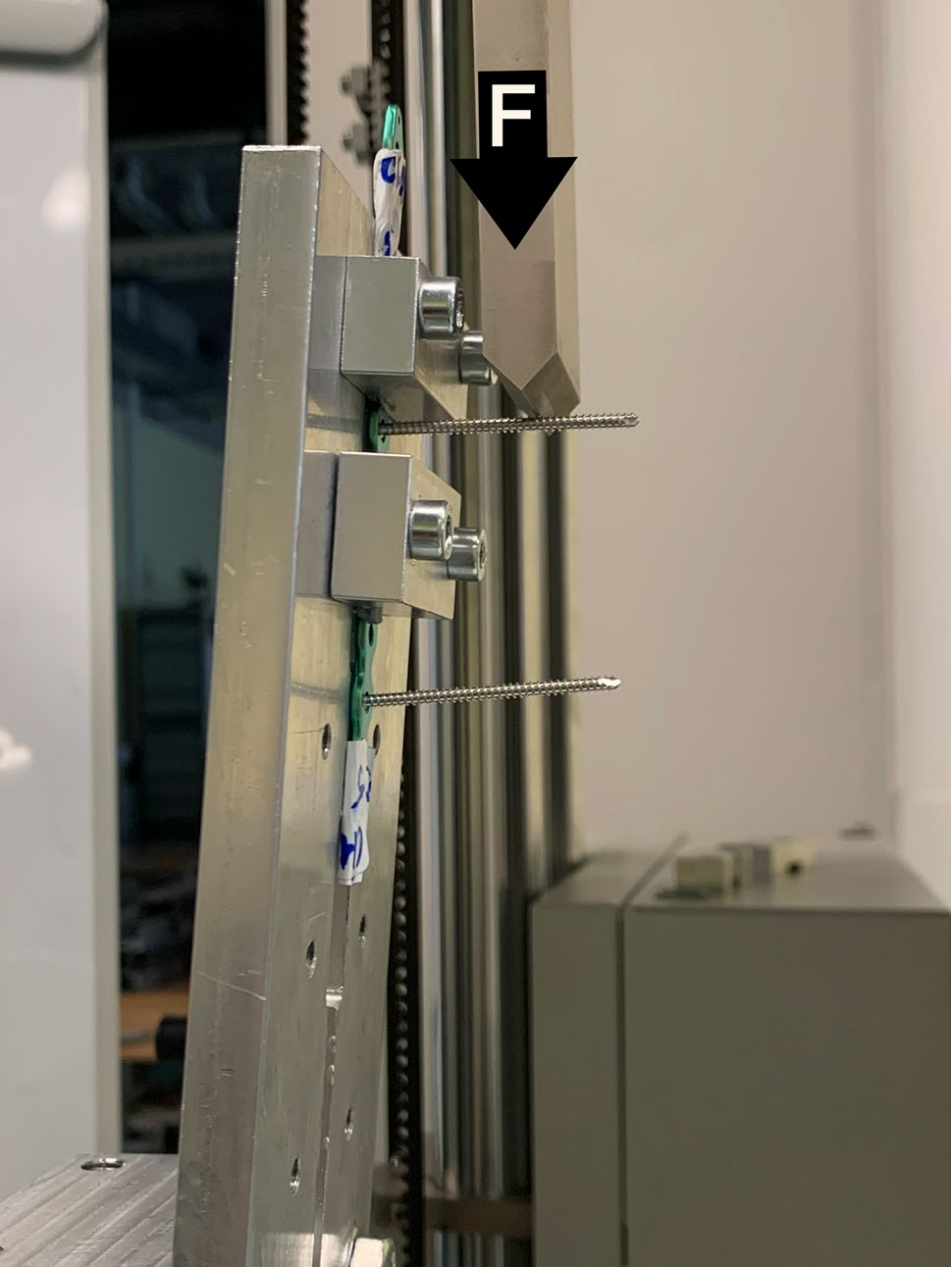
Set-up configuration.

A universal servohydraulic testing machine (ZWICK Z100/TL3S Zwick GmbH & Co. KG, Ulm, Germany) was employed to determine the bending strength of the polyaxial plate constructs, with results reported in Nm. Each plate was securely positioned in a custom-made jig designed to prevent any movement or bending when subjected to a perpendicular compressive load (Fig. 2). The load was consistently applied orthogonally to the screw axis for all insertion angles with a constant displacement of 1 mm/min at a distance of 21.6 mm from underneath the plate. We continued testing until screw failure, defined as a breakage or a rapid loss of over 50% of force. The bending moment was calculated by multiplying the force acting on the screw and that distance. Our focus was on determining the maximum bending moment just before the abrupt decrease caused by the failure of screw-plate threaded interface. The higher the bending strength of the construct, the higher its stability under bending. All measurements were performed following the adapted standards of ASTM, specifically ASTM Standard F384-17^19^.

### Statistics

Statistical analyses were performed using Mathematica 12 software (Wolfram Research, Inc., Oxfordshire, United Kingdom). The data were reported as mean ± standard deviation, statistical significance was set at p < 0.05.

One-way ANOVA was employed to assess the impact of the screw insertion method on both average thread engagement (ATE) and bending strength. The normality of the residuals was verified using the Shapiro–Wilk test. Post-hoc Tukey test was applied to determine whether there were any significant differences between the groups. Linear regression analysis was performed to determine the strengths of the correlation between ATE and bending strength.

## Results

**Figure 3:**
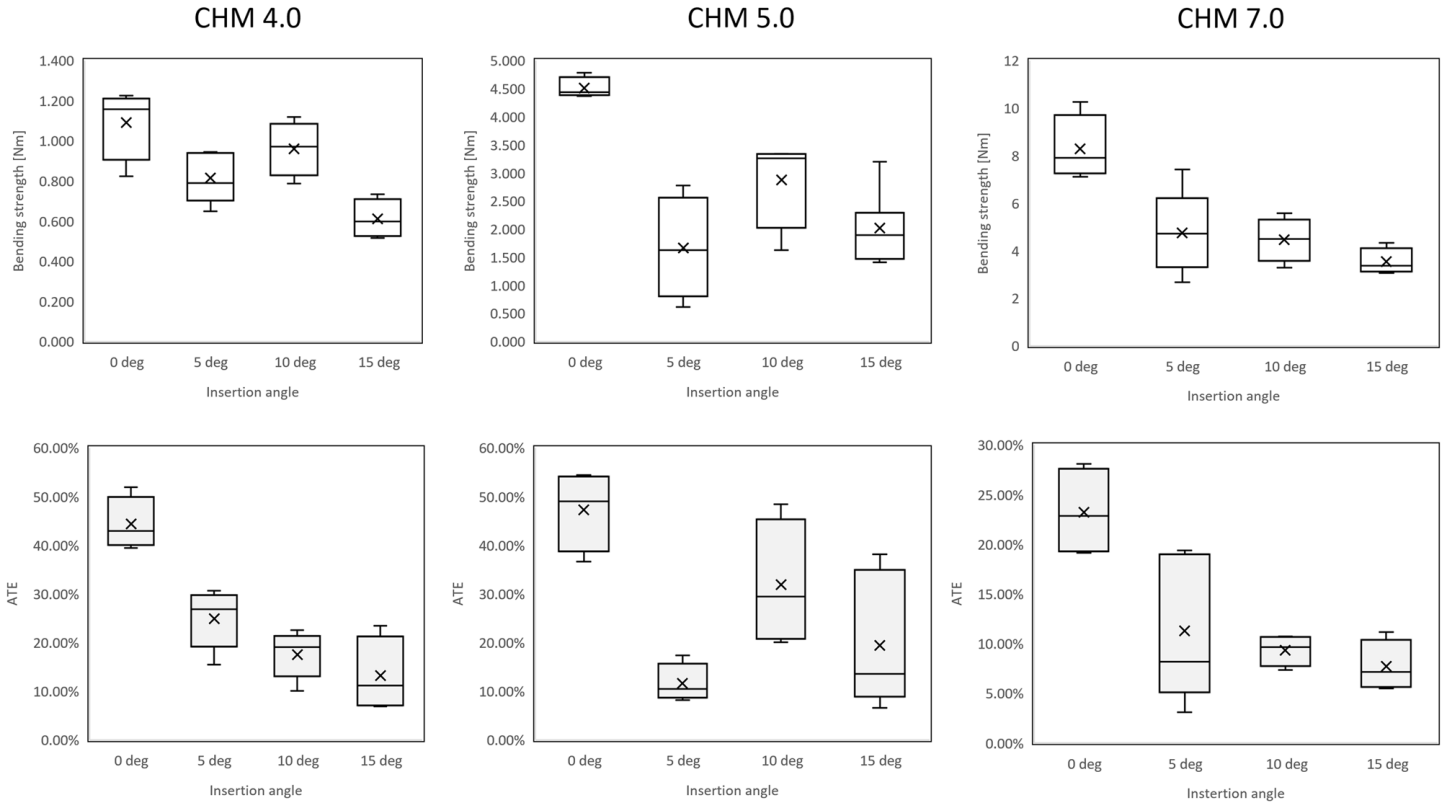
Box and whisker plots of bending strength (top row) and ATE (bottom row) for all analyzed angles and ChLP systems (columns).

Micro-CT analysis revealed that the placement of screws at different angles affected the average thread engagement, with the initial contact area highest for screws inserted at 0° and lowest for samples aligned at 15° in all analyzed systems. Statistical examination with the ANOVA test demonstrated that the angle of insertion was a statistically significant factor affecting ATE (p < 0.001, p = 0.0012, p = 0.0021 for 4.0, 5.0 and 7.0 systems, respectively) (Table 1, Fig. 3).

**Table 1:** The relative contact area between the screw head and plate hole for 4.0, 5.0 and 7.0 ChLP polyaxial systems.

System	Degree	No. samples	ATE
4.0	0	4	44.31% ± 5.41%^a^
	5	5	24.91% ± 6.03%^b^
	10	5	17.55% ± 4.8%^bc^
	15	4	13.18% ± 7.67%^c^
5.0	0	4	47.25% ± 8.23%^a^
	5	4	11.58% ± 3.98%^b^
	10	4	31.85% ± 13%^ab^
	15	7	19.38% ± 12.56%^b^
7.0	0	4	23.23% ± 5.03%^a^
	5	5	11.26% ± 7.29%^b^
	10	4	9.33% ± 1.58%^b^
	15	4	7.72% ± 2.55%^b^

Values shown are mean ± standard deviation. Common superscript letters within the locking system denote significant differences in ATE (p < 0.05).

**Figure 4:**
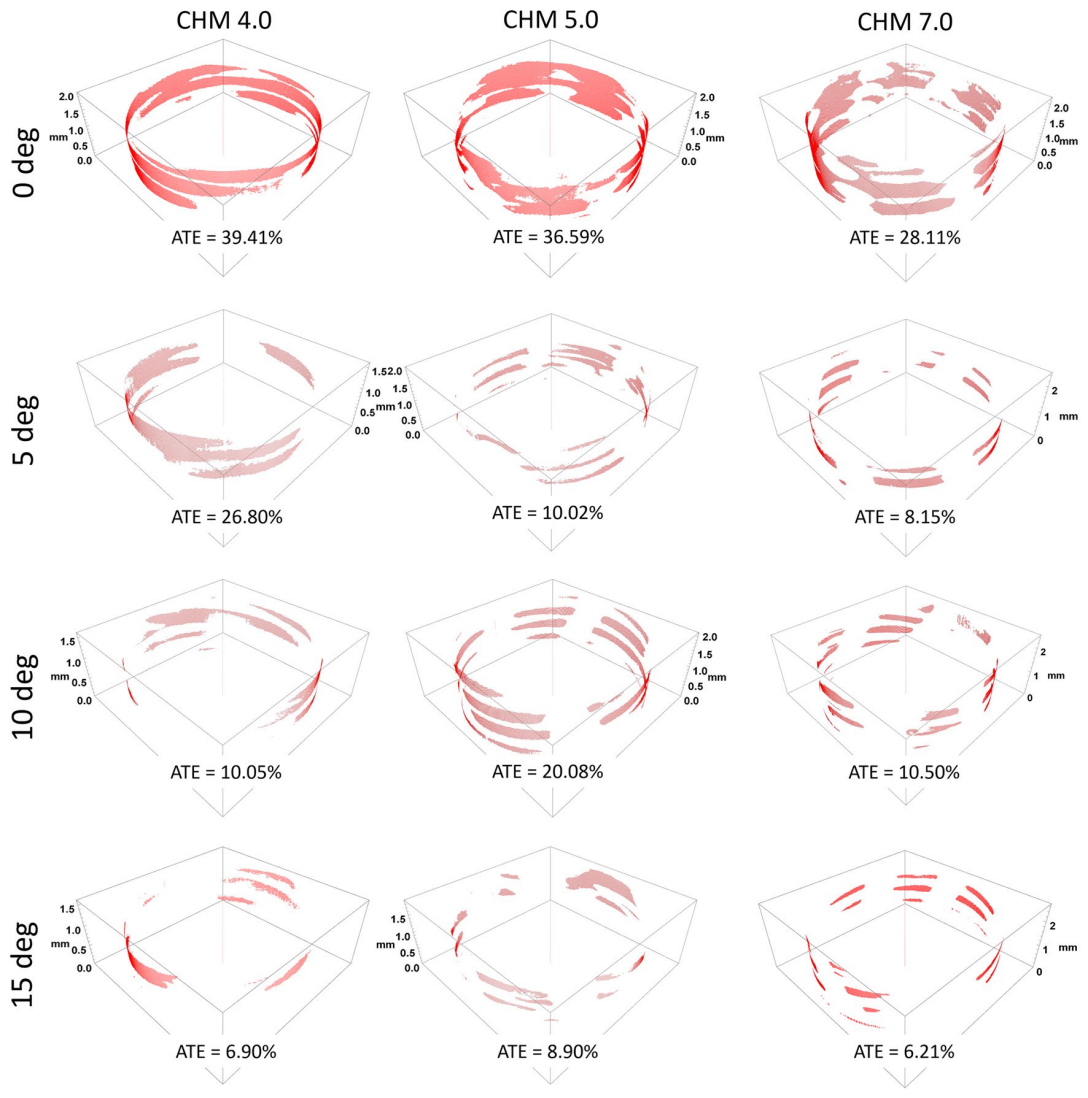
A visualization of thread engagement in screw-plate interfaces inserted at 0°, 5°, 10°, 15° angles (rows) for all three analyzed ChLP systems (columns).

Nevertheless, the post-hoc Tukey test revealed that, overall, screws inserted off-axis at angles ranging from 5° to 15° exhibited sufficient anchoring, with no statistically significant differences between each off-axis group (refer to Table 1 for details). Notably, the results of average thread engagement for the 5.0 ChLP system showed considerable variability in comparison to the 4.0 and 7.0 ChLP systems. Figure 4 presents a visualization of the screws placed at 0° to 15° off-axis, depicting the ATE.

The initial assessment involved a control group of 4.0 ChLP polyaxial screws inserted at 0°. The screws failed without relocation in the screw hole and rapid force loss, due to plastic deformation of the screw with macroscopic bending of the screw shaft. A stable construct could also be achieved at 5°, 10° and 15° angles, with all screws undergoing plastic deformation without a relocation in the screw hole. The 5.0 ChLP polyaxial screws inserted at 0° similarly exhibited failure marked by macroscopic bending of the screw shaft. Notably, the macroscopic failure of the screw head-plate hole interface was specific to the 5.0 ChLP system, resulting in identical failures for all screws inserted off-axis. This disfigurement was attributed to the plastic deformation of the titanium alloy used in the 5.0 ChLP plate.

**Figure 5:**
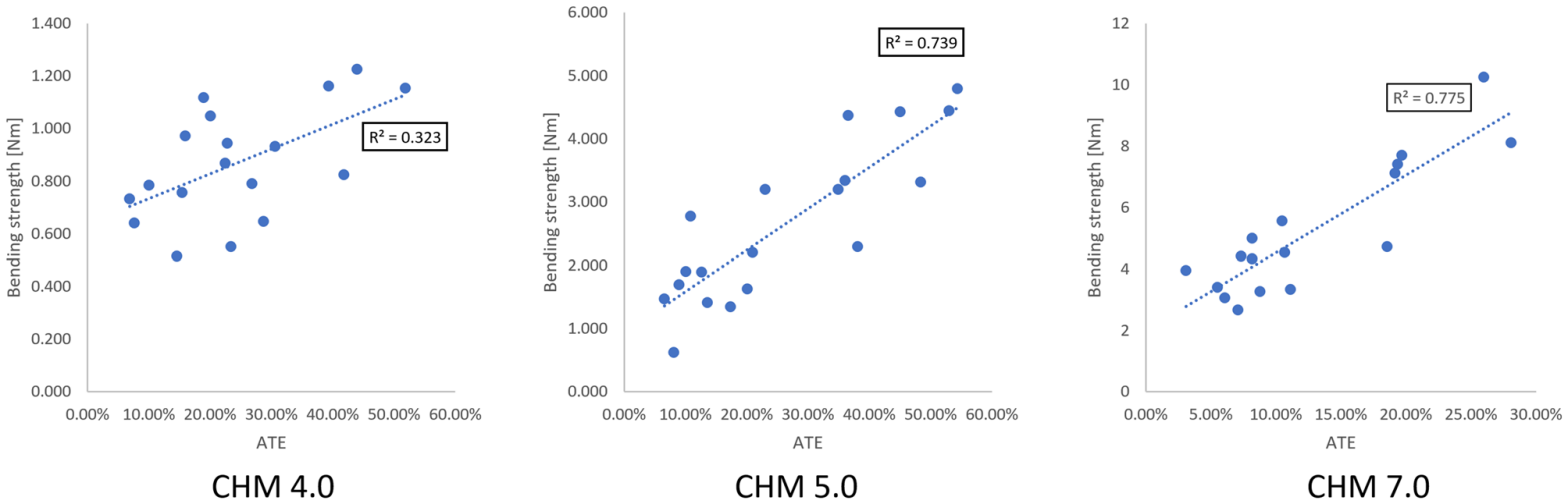
Bending strength of 4.0, 5.0, 7.0 ChLP system plates with polyaxial screws inserted at different angles with relation to thread engagement.

Similar to 4.0 and 5.0 ChLP systems, the 7.0 ChLP screws inserted at 0° experienced failure without relocation in the screw hole, with macroscopic breakage of the screw shaft observed in all cases. All 7.0 ChLP screws inserted off-axis failed at the screw-plate interface. Mechanical tests demonstrated that the most stable construct was achieved when screws were placed monoaxially, and its strength decreased when inserted off-axis (refer to Table 2 for details). Statistical analysis (ANOVA test) demonstrated that the bending strength is significantly impacted by the insertion method (p < 0.001) (Fig. 5). Interestingly, the post-hoc Tukey test revealed that generally the screws inserted off-axis between 5° to 15° exhibited bending strength with no statistically significant difference between each off-axis group. For the 5.0 and 7.0 ChLP systems, there was a strong correlation between bending strength and ATE (R2 > 0.7). In contrast, a weaker association between thread engagement and bending performance was noted for smaller ChLP system 4.0.

**Table 2:** Mechanical parameters of the 4.0, 5.0 and 7.0 ChLP polyaxial systems and angles tested.

System	Degree	No. samples	Bending strength [Nm]
4.0	0	4	1.09 ± 0.18^a^
	5	5	0.81 ± 0.13^bc^
	10	5	0.96 ± 0.13^ab^
	15	4	0.61 ± 0.1^c^
5.0	0	4	4.51 ± 0.19^a^
	5	4	1.66 ± 0.91^b^
	10	4	2.87 ± 0.83^b^
	15	7	2.02 ± 0.62^b^
7.0	0	4	8.29 ± 1.37^a^
	5	5	4.75 ± 1.74^b^
	10	4	4.45 ± 0.94^b^
	15	4	3.53 ± 0.56^b^

Values shown are mean ± standard deviation. Common superscript letters within the locking system denote significant differences in bending strength (p < 0.05).

## Discussion

Polyaxial plates have been developed to assist surgeons during challenging fracture fixation. These systems aim to mitigate the negative effects of cold welding, a consequence observed in previous monoaxial systems when screws were placed off-axis^28^. Kaab et al. described a substantial reduction in force of up to 68%, associated with improper insertion of monoaxial locking screws^8^. On the other hand, in the study conducted by Herbert-Davies et al., off-axis insertion of the monoaxial screw was associated with a reduction in load of up to 60%^28^.

Most studies focused on polyaxial plates conduct biomechanical tests utilizing fracture models or whole constructs with multiple screws inserted to mimic clinical standard clinical application^29–31^. Yet, several studies proved no statistically significant difference compared to traditional monoaxial fixations^14,31,32^. Investigations using synthetic bone constructs demonstrated similar stability between polyaxial and monoaxial plates^30^. However, composite models may not accurately replicate bone inhomogeneity, potentially affecting the performance of tested implants^14^. In contrast, other studies conducted on fracture gap models utilized different available cadaveric bones, including formalin-fixed and fresh frozen bone specimens with variable bone stock^33^. Although polyaxial locking screws offer advantageous properties, off-axis screw insertion comes with certain limitations^10^. Polyaxial interfaces, being non-standard threaded connections, rely heavily on the precision of screw insertion to maintain their strength^10^. Our study focuses on individual screw failure, diverging from the conventional approach of evaluating the entire construct. Few studies have investigated single polyaxial screw-plate interface^8,10,28^. However, caution is warranted when making direct comparisons of bending moments between different systems due to the varied range of performed tests, plate sizes, and the utilization of several types of locking mechanisms.

In our study, we employed micro-CT to quantify thread-to-thread engagement, providing insights into the locking mechanism of polyaxial screws based on the varying insertion angles. Our data revealed that nearly 50% of thread-to-thread engagement was achievable when polyaxial screws were inserted perpendicularly to the plate. However, in the most critical scenario – a 15° off-axis placement of the screws – there was a noteworthy reduction of ATE, dropping to as little as 13%, 19% and 7% for the 4.0, 5.0 and 7.0 ChLP systems, respectively. The novelty of micro-CT analysis at screw-plate interface bridges a gap in the literature by expanding data to include the initial contact area between the threads. As of now, direct comparisons with other available systems used in human subjects are not possible. To our knowledge, micro-CT quantification methods have only been applied to veterinary in previous studies^26^.

The results of our non-destructive analysis aligned with the mechanical parameters of ChLP systems. The bending strength of all analyzed systems was affected by the insertion angle, revealing a significant reduction of 44%, 55% and 57% for the 4.0, 5.0 and 7.0 ChLP systems, respectively, when inserted at the maximum angle of 15°. A study of Herbert-Davies et al. found similar reductions in maximal bending force for Stryker VariAX 3.5 mm and the Peri-Loc 3, 5 mm, irrespective of the different locking mechanisms^28^. The maximal acceptable insertion of 15° was associated with 45% and 34% reduction of force, respectively^28^.

In contrast, Zimmer NCB plates showed enhanced maximal bending forces with off-axis insertion, albeit with large standard deviations. However, the initial engagement of the threads and end cap surface contact between the screw head at 0° and 15° were not assessed^28^. Our results differ from these findings, as a significant reduction of force was achieved beyond 10° of insertion for all analyzed systems. Thus the authors concluded that the actual safe zone is limited a 10° insertion of the screw^28^. Our post-hoc Tukey test revealed no significant differences in bending moment between each off-axis group of ChM manufacturer plates. Tidwell et al. demonstrated that off-axis insertion was almost linearly correlated with bending force loss^10^. However, those findings were limited to Synthes polyaxial plate^10^. Mehling et al. presented different conclusions, noting that the comparable cut-in design of polyaxial interfaces had the highest ultimate strength at 10° to 15° of screw insertion^31^. Meanwhile, the point-loading thread-in interface exhibited the highest strength at a maximum of 5° of screw insertion^31^. Hoffemeier et al. similarly found that the point-loading thread-in design had the highest bending strength at the neutral insertion of the polyaxial screws^20^.

Despite the considerable utility of polyaxial systems, particularly in the management of periprosthetic or periarticular fractures, there is a scarcity of studies on the mechanical performance of these plates^15,34^. Previous research proved that mechanical performance of different designs can vary significantly, depending on the angle of insertion^11^. Historically, the failure model of monoaxial implants typically resulted from the pull-out of the bone, due to poor bone purchase^35^. Hardware complications at the screw plate interface were rare^35^. However, in cases involving the lower extremity and prior to radiographic union, certain polyaxial mechanisms could produce a lower-strength construct due to screw disengagement in the plate-screw interface.

This study has several limitations, attributed primarily to the ex vivo testing, which cannot reflect conditions experienced by the plate in reduced fracture site. Moreover, our analysis focuses on individual locking mechanisms rather than the entire construct. Additionally, we did not analyze bone-screw interface, which could transmit loading different than that encountered in mechanical tests^36^. Another potential limitation of this study is the lack of consideration for the influence of the coating technology used for the hardware^37^.

## Conclusions

In terms of the impact of screw locking angles, the thread-in polyaxial mechanism exhibits comparable stability when screws are placed off-axis. However, there is a particular decrease of force that influences stability when compared to monoaxial placement. Therefore, angulations of the screws in these systems should be considered as an option rather than a routine practice.

## Data Availability

The data that support the findings of this study are available on request from the corresponding author [J.G.].
